# A new in vivo model using a dorsal skinfold chamber to investigate microcirculation and angiogenesis in diabetic wounds

**DOI:** 10.3205/iprs000088

**Published:** 2016-02-18

**Authors:** Stefan Langer, Christian Beescho, Andrej Ring, Olivia Dorfmann, Hans Ulrich Steinau, Nick Spindler

**Affiliations:** 1 Department of Plastic, Esthetic and Special Hand Surgery, University Hospital Leipzig, Germany; 2 Department of Plastic Surgery and Severe Burns, University Hospital Bergmannsheil, Ruhr University Bochum, Germany; 3 Department of Trauma, University Hospital Essen, Germany

**Keywords:** skinfold chamber, microcirculation, angiogenesis, diabetic wounds

## Abstract

**Introduction:** Diabetes mellitus describes a dysregulation of glucose metabolism due to improper insulin secretion, reduced insulin efficacy or both. It is a well-known fact that diabetic patients are likely to suffer from impaired wound healing, as diabetes strongly affects tissue angiogenesis. Until now, no satisfying in vivo murine model has been established to analyze the dynamics of angiogenesis during diabetic wound healing. To help understand the pathophysiology of diabetes and its effect on angiogenesis, a novel in vivo murine model was established using the skinfold chamber in mice.

**Materials and Methods: **Mutant diabetic mice (db; *BKS.Cg-m+/+Lepr^db^/J*), wildtype mice (*dock7Lepr^db^+/+m*) and laboratory BALB/c mice were examined. They were kept in single cages with access to laboratory chow with an 12/12 hour day/night circle. Lesions of the panniculus muscle (Ø 2 mm) were created in the center of the transparent window chamber and the subsequent muscular wound healing was then observed for a period of 22 days. Important analytic parameters included vessel diameter, red blood cell velocity, vascular permeability, and leakage of muscle capillaries and post capillary venules. The key parameters were functional capillary density (FCD) and angiogenesis positive area (APA).

**Results:** We established a model which allows high resolution in vivo imaging of functional angiogenesis in diabetic wounds. As expected, db mice showed impaired wound closure (day 22) compared to wounds of BALB/c or WT mice (day 15). FCD was lower in diabetic mice compared to WT and BALB/c during the entire observation period. The dynamics of angiogenesis also decreased in db mice, as reflected by the lowest APA levels. Significant variations in the skin buildup were observed, with the greatest skin depth in db mice. Furthermore, in db mice, the dermis:subcutaneous ratio was highly shifted towards the subcutaneous layers as opposed to WT or BALB/c mice.

**Conclusion:** Using this new in vivo model of the skinfold chamber, it was possible to analyze and quantify microangiopathical changes which are essential for a better understanding of the pathophysiology of disturbed wound healing. Research in microcirculation is important to display perfusion in wounds versus healthy tissue. Using our model, we were able to compare wound healing in diabetic and healthy mice. We were also able to objectively analyze perfusion in wound edges and compare microcirculatory parameters. This model may be well suited to augment different therapeutic options.

## Zusammenfassung

**Einleitung:** Diabetes Mellitus beschreibt die Dysregulation des Glukosemetabolismus auf Grund von pathologischer Insulin-Sekretion, reduzierter Insulin-Effizienz oder beidem. Es ist hinreichend bekannt, dass Patienten mit einem Diabetes mellitus an verlängerter Wundheilung leiden, da die Weichteilangiogenese hierdurch massiv beeinflusst wird. Bis zum jetzigen Zeitpunkt ist kein befriedigendes in vivo murine Modell etabliert, um die Dynamik der Angiogenese während einer diabetischen Wundheilung zu untersuchen. Um die pathophysiologischen Abläufe des Diabetes und seinen Einfluss auf die Angiogenese besser verstehen zu können, wurde ein neues in vivo murine Modell entwickelt bei welchem mittels einer Hautkammer die Veränderungen bei Mäusen sichtbar gemacht werden.

**Material und Methoden:** Diabetische Mäuse (db; *BKS.Cg-m+/+Lepr^db^/J*), Wildtyp Mäuse (*dock7Lepr^db^+/+m*) sowie BALB/c Labormäuse wurden hierzu untersucht. Diese wurden in Einzelkäfigen gehalten mit selbstständigem Futterzugang in einem 12-stündigen Tag- und Nachtrhythmus. Muskelläsionen von 2 mm Durchmesser wurden im Zentrum des Hautkammerfensters gesetzt. Hierauf hin wurde die Wundheilung über einen Zeitraum von 22 Tagen verfolgt. Wichtige analytische Daten wie Gefäßdurchmesser, Fließgeschwindgkeit, Gefäßpermeabilität sowie das Kapillar-Leck von Muskelkapillaren sowie post-kapilläre Venolen konnten hierbei erhoben werden. Schlüsselparameter waren die functional capillary density (FCD) und die angiogenesis positive areas (APA).

**Ergebnisse:** Wir haben ein Wundmodell etabliert, welches hoch aufgelöste in vivo Aufnahmen der funktionellen Angiogenese der diabetischen Wunde ermöglicht. Wie angenommen zeigten die db Mäuse eine gestörte Wundheilung (22. Tag) verglichen mit den Wunden einer BALB/c oder WT Maus (15. Tag). FCD war über den gesamten Verlauf bei den diabetischen Mäusen niedriger als bei WT oder BALB/c. Die Dynamik der Angiogenese nahm bei diabetischen Mäusen ab, was der niedrige APA Level widerspiegelt. Signifikante Variationen im Hautaufbau wurden beobachtet, mit der stärksten Hautdicke in der diabetischen Maus. Viel mehr noch änderte sich der Dermis-Subkutan Ratio zu Gunsten des subkutanen Gewebes im Gegensatz zu WT oder BALB/c Mäusen.

**Zusammenfassung:** Unter Verwendung des beschriebenen Hautkammer-Modells konnten die mikroangiopathischen Veränderungen, welche essentiell für das Verständnis der Patholphysiologie der veränderten Wundheilung sind, untersucht und quantifiziert werden. Untersuchungen der Mikrozirkulation konnten die Unterschiede der Perfusion von gesundem zu erkranktem Gewebe darstellen. Mittels unseres Hautkammer-Modells konnten wir die Wundheilung in diabetischen Mäusen darstellen. Des Weiteren konnten objektive Analysen zu Durchblutung der Wundränder erhoben und mit Parametern von gesunden Individuen verglichen werden.

Unser Hautkammermodell ist daher gut zur Untermauerung verschiedenster therapeutischer Ansätze geeignet.

## Introduction

Diabetes mellitus is defined as a dysregulation of glucose metabolism due to improper insulin secretion, reduced insulin efficacy or both [1]. Diabetes is a common lifelong health condition, which plays an important role in angiogenesis and tissue healing. Tissue healing is a physiological process where reperfusion and angiogenesis of capillaries and soft tissue support wound closure [2]. It is a well-known fact that diabetic patients are likely to suffer from impaired wound healing. Dysregulation in angiogenesis is closely correlated with impaired wound healing. 

The capacity to react to hypoxic conditions with adaptive reconstruction of vessels is limited in diabetic patients. When examining the skin of patients with acute hyperinsulinemia (with or without hyperglycemia), no correlation to vessel permeability, hemodynamics, or parameters of endothelial dysfunction could be identified. It remains unclear whether reduced insulin sensitivity is responsible for microcirculatory disturbances [3]. Increased blood viscosity is another pathological factor in diabetics. Moreover, the aggregation of erythrocytes is increased, as is their inability to deform themselves when passing through the capillary system [4], [5], [6], [7], [8]. Unfortunately, there are no in vivo murine models available which would permit close analysis of wound microcirculation in vivo. The aim of the study was to establish a novel mouse model which allows for visualization of microcirculatory disturbances which play a key role in the pathophysiology of diabetic wound healing.

## Materials and methods

Diabetic mice from the strain *BKS.Cg-m+/+Lepr^db^/J* with a homozygous mutation in the leptin receptor were compared to a control group in terms of macrocirculatory and microcirculatory characteristics. Leptin is an important regulator of the appetite center of the brain, and therefore, diabetic leptin-mutated *BKS.Cg-m+/+Lepr^db^/J* mice suffer from polypghagia, polydipsia, and polyuria. After four to eight weeks, the *BKS.Cg-m+/+Leprdb/J* mice showed increased blood sugar levels. 

The control group included wildtype mice of the strain *dock7 Lepr^db^+/+m *with the misty mutation. They exhibited normal weight, blood sugar and plasma insulin, had an increased metabolic efficiency, and were non-diabetic. We also used another control group consisting of 8-week-old BALB/c female laboratory mice All mice were weighed at baseline and kept in single housing throughout the experiments. 

The model of a transparent skinfold chamber is an established in vivo system that enables studies on angiogenesis. By refining the skinfold chamber through microsurgical implantation techniques, this model could be applied in mice for research purposes. Along with the observation of healthy and pathological tissue it also was possible to examine transplants, tissue replacement substances such as surgical mesh grafts, tissue engineering, and topically/systemically applied medication [9], [10], [11]. The vascularized striated muscle is well represented in the skinfold chamber [12], [13], [14], [15] and can be directly visualized when combined with epifluorescence microscopy.

We implanted the skinfold chamber and created a lesion in the dorsal skin muscle (m. panniculus carnosus) using skin punches (disposable biopsy punch, 2 mm; Stiefel, Germany). A circumscribed circular edge was established and the wound was covered with the coverslip of the skinfold chamber. No air was enclosed and a snap ring fixed the coverslip in place. All mice received a skinfold chamber and the same standardized lesion. 

## Results

After the chamber implantation, we examined primary wound healing of the intramuscular lesion for a period of 22 days. The mice were immobilized using a PAC (polyacrylate) tube with a frontal valve opening bearing a slit for the skinfold chamber, which was adapted to the exact girth of each mouse. The tube could be fixed on a tablet for microscopic observation.

**Figure 1:**
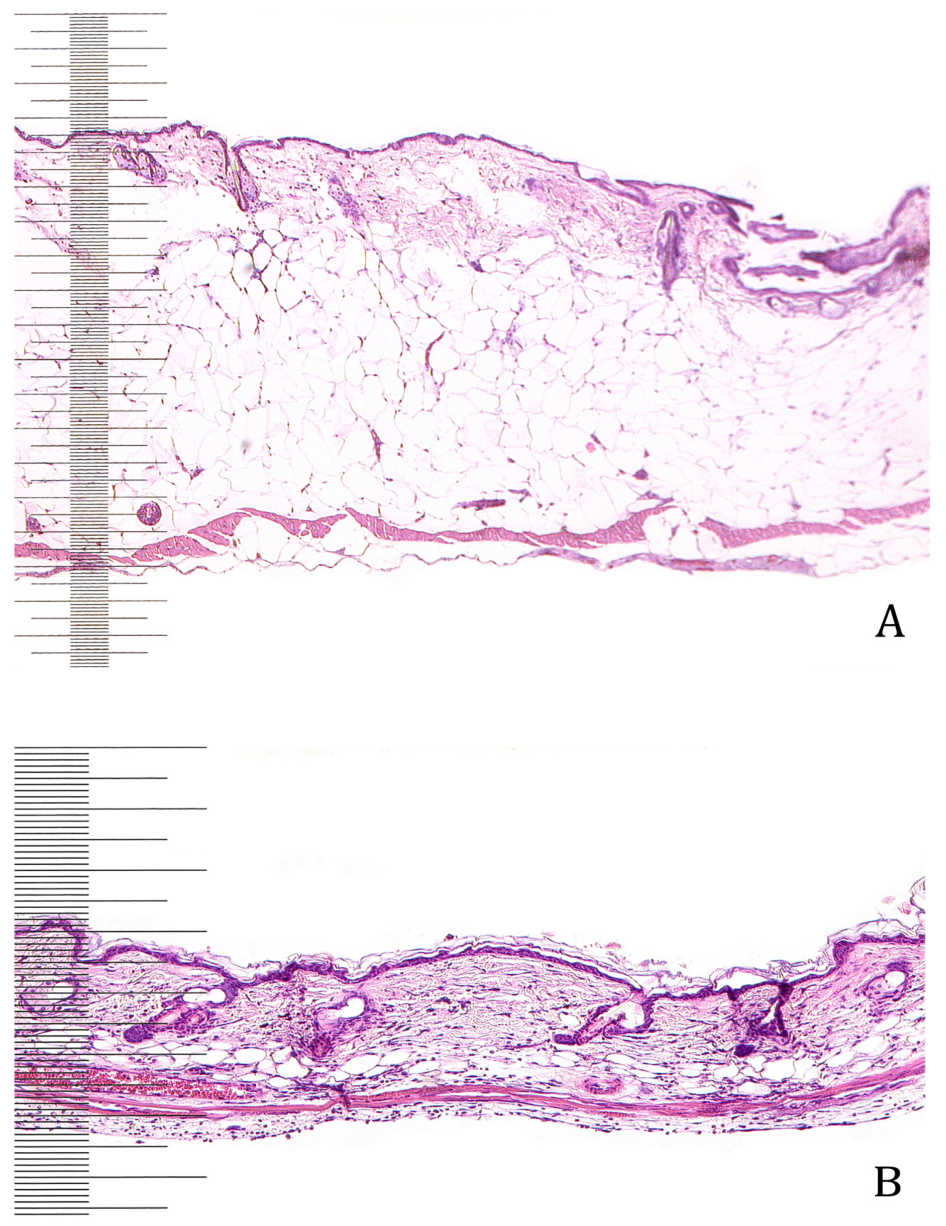
Hematoxylin and eosin staining of db/db mice (A) and wildtype mice (B). The differences in skin thickness are apparent.

**Figure 2:**
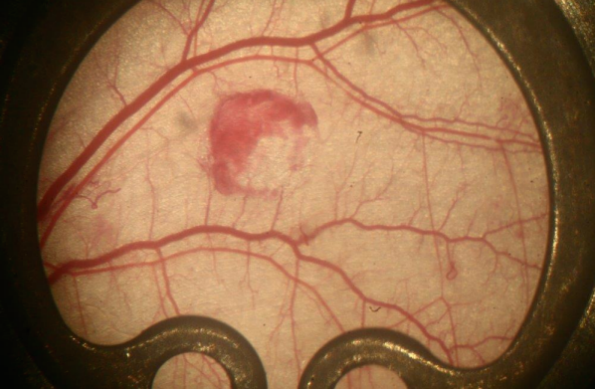
Circular wound of the skin muscle layer on the day of wounding

**Figure 3:**
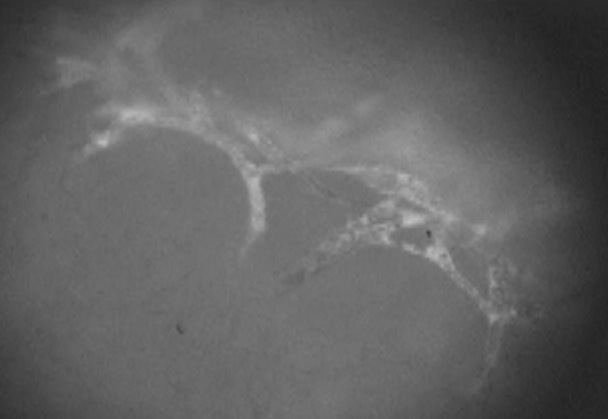
Vessel sprouts visualized using FITC-Dextran as plasma enhancement. Dynamics of vessel development can be analyzed repeatedly over 3 weeks using this model. (bar=10 µm)

A digital camera was used to take macroscopic pictures, which served as the basis for the subsequent calculations of the wound area. The microcirculation was documented from 24 hours to 11 days after chamber implantation (see Figure 1 (Fig. 1), Figure 2 (Fig. 2), Figure 3 (Fig. 3)). We acquired additional video data by using 400X magnification to film the edges of the wound and used the computer-assisted image analysis program CapImage (Version 7.4, Dr. Zeintl Software; Heidelberg, Germany). Using this program, we also defined parameters such as vessel diameter (µm), midstream red blood cell velocity (RBCV; mm/s), leakage of muscle capillaries and post capillary venules given by the ratio of fluorescence inside vessel vs outside vessel (I_e_/I_i_). We also determined the functional capillary density (FCD), meaning red blood cell filled capillaries (mm/mm^2^). 

In postcapillary venules, we determined the amount of rolling leucocytes on the endothelium. Angiogenesis positive areas (APA) were counted manually (number/area).

The depth of the dorsal skin varied greatly among our mouse groups. In BALB/c mice, the dorsal skin depth was 370 µm, whereas in *dock7Lepr^db^+/+m *mice it was 500 µm. The skin was thickest in db/db mice with values ranging from 1000 to 1400 µm. When comparing the single skin layers among mouse strains, we discovered that the epithelia did not differ significantly. The dermis of db/db mice had a thickness of 250 µm and the subcutaneous layer a thickness of 500 to 1000 µm, providing a dermis: subcutaneous ratio ranging from 1:2 to 1:4. The dermis of BALB/c mice showed a regular and parallel pattern and the thickness was 200 µm. The subcutaneous layer was only 50 µm thick, which leads to a dermis: subcutaneous ratio of 4:1. In *dock7Lepr^db^+/+m *mice, the dermis had a thickness of 200 µm, comparable to BALB/c mice; however, the subcutaneous layer was much thicker than in BALB/c mice, with values ranging from 150–200 µm. This resulted in a dermis: subcutaneous ratio of 1:1. The dorsal skin muscle was very compact in wildtype mice, with a thickness ranging from 100 to 150 µm. In BALB/c mice, this muscle was only 30 µm thick. In both control groups, the muscle cells follow a regular, parallel and organized pattern. However, the skin muscle in db/db mice (which is 60 µm thick) shows less compact and poorly organized layers. 

## Discussion

The skinfold chamber offers the advantage of possible continuous examination and analysis. By fixing the PAC tube on a tablet, it was possible to horizontally position the skinfold chamber for examination using photo- and intravital microscopy. These techniques enabled analysis and quantification of microangiopathical changes, which is essential for understanding the pathophysiology of disturbed wound healing. Research in microcirculation is important to display perfusion in wounds and healthy tissue. Skin perfusion largely depends on the body part. Thus, due to its anatomical localization, striated muscle shows far less fluctuation. Using our in vivo wound healing model, we were able to compare wound healing in diabetic and healthy mice. Our model also enabled us to objectively analyze perfusion within wound edges and compare microcirculatory parameters. In addition, this model offers the opportunity for systemic or topical therapeutic intervention. The influence of extracorporeal shock wave therapy (ECSW) on wound healing has already been successfully examined by our research group. The analytic spectrum will be extended.

Mice make excellent models to study in vivo wound healing, angiogenesis and neovascularization due to their high genomic resemblance to the human genome. *BKS.Cg-m+/+Lepr^db^/J* mice are especially suitable for analyzing diabetic wound healing, as they show a homozygous mutation in the leptin receptor. These mice suffer from polypghagia, polydipsia, and polyuria and exhibit the same characteristics as human diabetics. Research conditions can be modified, as different strains of mice may be used to allow the study of genetic variation. In addition to congenitally inbred stems such as BALB/c or C57B1/6 [16], [17], the transparent skinfold chamber can also be used on various genetically modified mice, such as naked mice [18], SCID mice [19], specific knock-out mice [20], [21] or transgenic mice [22]. The skinfold chamber is an ideal model for mice as opposed to other rodents [23], [24]. 

As mentioned above, we compared coagulation time among different mouse strains during and after surgery while using microsurgical swabs to staunch the flow of blood. Coagulation in *BKS.Cg-m+/+Lepr^db^/J *mice was much slower and required more time to be stopped. Only after multiple saline rinses and applying pressure to the tissue was bleeding controlled. Henry et al. confirm that *BKS.Cg-m+/+Lepr^db^/J* mice show a prolonged coagulation, which reflects a restricted ADP-dependent thromobocytic aggregation. This is an interesting observation, as type 2 diabetics generally show hypercoagulation associated with myocardial infarcts and stroke due to blood clots in vessels [25].

Prior to implanting the skinfold chamber, the nine week old *BKS.Cg-m+/+Lepr^db^/J* mice weighed 36.99 ± 0.91 g, matching the average body weight of 38 g ± SEMxxscanning electron microscope [26]. After the chamber implantation and creation of the wound, the body weight of the mice decreased by 2%. Other studies even describe weight loss of up to 15%, which can be attributed to chamber implantation [27], [28].

Nine-week-old dock7 Leprdb+/+m mice weighed only 24.57 ± 0.59 g. It is also known that high leptin sensitivity keeps animals thin and leptin resistance/lack of leptin leads to obesity. We suspect that the mutation in the leptin receptor in *BKS.Cg-m+/+Lepr^db^/J* mice caused the difference in weight of diabetic versus WT mice. Among all groups, BALB/c mice showed the lowest body weight with an average of 19–21 g [29].

## Conclusions

The possibilities in research are manifold with the chamber model. Along with the observation of healthy and pathological wound tissue [30], the skinfold chamber offers the tremendous advantage of therapeutic intervention, such as topical or systemic application of medication or application of growth factors (e.g., non viral genetic transfer). Other substances such as transplants, implants and tissue replacement materials may be equally applied [31], [32], [33], [34], [35]. In the past, the skinfold chamber model has been used as a bioreactor for in vivo visualization of transplants or in ischemic reperfusion. Vascularized striated muscle is well represented in the skinfold chamber [36] and can be directly visualized when combined with intra-vital epifluorescence microscopy. 

The combination of the skinfold chamber with a transparent window allows continuous observation of the surgical muscle lesion and the daily assessment of wound size. The possibility of intra-vital microscopy is a novelty in a murine model. The chamber model is especially valuable for precise detection of microcirculatory disturbances. To date, no other model comparable to the in vivo skinfold chamber exists and the model presented here is very suitable for long term quantitative analysis of wound healing in diabetic wounds. 

## Notes

### Competing interests

The authors herewith certify that there is no financial or proprietary interest in the subjected matter or materials discussed in this manuscript.

### Ethical Standards

Animal studies have been approved by the ethics committee of University of Bochum (AZ 8.87-503709135) and have therefore been performed in accordance with the ethical standards set forth in the 1964 Declaration of Helsinki and its later amendments. The manuscript does not contain clinical studies or patient data.
